# The Role of T Cell Receptor Signaling in the Development of Type 1 Diabetes

**DOI:** 10.3389/fimmu.2020.615371

**Published:** 2021-02-02

**Authors:** Matthew Clark, Charles J. Kroger, Qi Ke, Roland M. Tisch

**Affiliations:** ^1^ Department of Microbiology and Immunology, University of North Carolina at Chapel Hill, Chapel Hill, NC, United States; ^2^ Lineberger Comprehensive Cancer Center, University of North Carolina at Chapel Hill, Chapel Hill, NC, United States

**Keywords:** autoimmunity, diabetes, immunoregulation, T cell receptor (TCR) signaling, T cell differentiation

## Abstract

T cell receptor (TCR) signaling influences multiple aspects of CD4^+^ and CD8^+^ T cell immunobiology including thymic development, peripheral homeostasis, effector subset differentiation/function, and memory formation. Additional T cell signaling cues triggered by co-stimulatory molecules and cytokines also affect TCR signaling duration, as well as accessory pathways that further shape a T cell response. Type 1 diabetes (T1D) is a T cell-driven autoimmune disease targeting the insulin producing β cells in the pancreas. Evidence indicates that dysregulated TCR signaling events in T1D impact the efficacy of central and peripheral tolerance-inducing mechanisms. In this review, we will discuss how the strength and nature of TCR signaling events influence the development of self-reactive T cells and drive the progression of T1D through effects on T cell gene expression, lineage commitment, and maintenance of pathogenic anti-self T cell effector function.

## Introduction

T cell-driven autoimmune diseases are heterogeneous and complex, typically leading to chronic organ-specific inflammation and tissue damage. Type 1 diabetes (T1D) is a prominent example of a T cell-mediated autoimmune disease. T1D is characterized by the destruction and/or dysregulation of the insulin producing β cells found in the pancreatic islets of Langerhans (1–5). Typically, β cell autoimmunity progresses for a number of years prior to clinical diagnosis of T1D (6, 7). The autoimmune response is heterogeneous marked by the disparate onset between childhood and adult diabetes (7–22). Inflammation of the islets, termed insulitis, involves infiltration of CD4^+^ and CD8^+^ T cells, B cells, dendritic cells (DC) and macrophages, as well as production of islet-specific autoantibodies (7, 9–18, 23, 24). When 80%–90% of β cell mass is rendered nonfunctional, hyperglycemia is established, and overt diabetes diagnosed. Indirect evidence indicates that human T1D is driven by T cells (9–18). For instance, the strongest genetic risk factor for T1D is associated with specific alleles of HLA class II and class I molecules (25–30). CD4^+^ and particularly CD8^+^ T cells are typically detected infiltrating the islets of T1D subjects (13, 25–28). Furthermore, the more aggressive childhood versus adult T1D onset is characterized by an expanded effector T cell (Teff) response to β cell autoantigens, such as proinsulin and insulin (11). Moreover, in non-obese diabetic (NOD) mice, a spontaneous model of T1D, CD4^+^ and CD8^+^ T cells are essential for β cell destruction, and respond to similar β cell autoantigens (5, 20, 31, 32). The NOD mouse model has been integral for studying the immunology of T1D, and investigating immunotherapies. The diabetogenic response in NOD mice, however, is considered to represent only one scenario of the heterogenous disease process in human T1D. For instance, examples exist suggesting that human T1D can progress in a T cell-independent manner. Nevertheless, CD4^+^ and CD8^+^ T cells are thought to be the primary drivers of β cell autoimmunity in most cases of human T1D (13, 33, 34).

The tissue-specificity and progression of T cell-mediated autoimmunity is dictated in part by the repertoire of T cell receptors (TCR) expressed by pathogenic Teff (18). The TCR repertoire in general is inherently self-reactive, and T cells recognizing self-peptides are detected in healthy individuals (11, 35–38). Typically, autoreactive T cells are tightly regulated by both central and peripheral tolerance-inducing mechanisms that prevent autoimmune-mediated pathology. However, in susceptible individuals, aberrant self-tolerance allows for the development, expansion and differentiation of autoreactive Teff that mediate pathology. In T1D numerous genetic variants are associated with disease susceptibility and resistance (27–30, 39–44). Notably, several of these insulin-dependent diabetes mellitus (IDDM) loci contain candidate genes that are involved with various aspects T cell immunobiology (45–50). The result is preferential skewing largely toward differentiation and expansion of β cell-specific type 1 CD4^+^ (e.g. T helper 1 (Th1)) and CD8^+^ (e.g. type 1 CD8^+^ (Tc1)) Teff, marked by expression of IFN*γ* and other proinflammatory cytokines (11, 51–53). Additional Teff subsets have been associated with driving β cell autoimmunity (51, 54–56). For example, the frequency of peripheral blood T follicular helper cells (Tfh) in human T1D patients correlates with increasing autoantibody production and reductions in C-peptide levels (57, 58). Furthermore, the *IL2*/*IL21* genomic region has also been identified as a risk factor in genome-wide association studies of human T1D subjects (41). Interestingly, clinical responsiveness of T1D patients to Abatacept treatment, which entails blockade of the CD80 and CD86 costimulatory molecules, directly correlates with a reduced pool of functional Tfh (59). These studies highlight Tfh as a key predictor of T1D disease progression (59). In addition, NOD mice fail to develop diabetes in the absence of IL-21, further suggesting that Tfh as well as Th17 are important contributors to β cell autoimmunity (56, 60–66).

Aberrant immunoregulation also contributes to the differentiation and expansion of pathogenic Teff in T1D (67, 68). In general, both natural and induced immunoregulatory CD4^+^ T cells expressing the forkhead box P3 protein transcription factor (nFoxp3^+^Treg and iFoxp3^+^Treg, respectively) play a critical role in suppressing autoimmunity (69–76). In human and NOD mouse T1D, reports have described aberrant maintenance, fitness and/or function of the Foxp3^+^Treg pool (54, 77–91). In addition, intrinsic defects within human and murine T1D Teff promote resistance to Foxp3^+^Treg-mediated suppression (77, 92).

The nature of a Teff response is influenced by multiple stimuli including TCR signal strength and duration, and/or the availability of co-stimulatory molecules and cytokines provided by antigen presenting cells (APC) (93, 94). Typically, strong TCR signaling is associated with a Th1 and Tc1 response regulated by the transcription factor T-bet. Since islet resident T cells largely exhibit a type 1 phenotype, this suggests that TCR signaling events favor differentiation of proinflammatory Teff (51, 52). In this review, we will discuss how key TCR signaling events in human T1D patients and the NOD mouse alters T cell development in the thymus that favors an autoreactive TCR repertoire, and how dysregulation of TCR signaling in the periphery imprints a proinflammatory phenotype in β cell-specific Teff that drives pancreatic islet damage.

## Thymic Origins of T Cell Receptor-Driven β Cell-Specific Autoimmunity

### Thymic Selection Events Shape the Anti-Self T Cell Receptor Repertoire

TCR signaling plays a pinnacle role in regulating T cell homeostasis, activation, expansion and effector function upon recognition of cognate foreign- or self-antigens. The specificity and properties of the TCR repertoire are established *via* selection events ongoing in the thymus (95–100). Positive selection occurring in the thymus cortex establishes a functional pool of TCR that bind self-peptide-MHC class I or II complexes. Cortical thymic epithelial cells (cTEC) mediate positive selection by presenting alternatively processed self-peptides to double positive thymocytes (DP), characterized in part by expression of the TCR, and both CD4 and CD8 co-receptors. Sufficient binding of TCR to self-peptide-MHC class I or class II molecules results in signaling events that promote DP survival and differentiation into CD8^+^ or CD4^+^ single positive thymocytes (SP), respectively. In the absence of TCR stimulation, DP undergo apoptosis. Since self-peptides drive positive selection, all functional TCR exhibit some degree of self-reactivity.

Positively selected SP migrate from the thymic cortex into the medulla to undergo negative selection. The thymic medulla is populated by medullary TEC (mTEC), DC, and B cells which present peripheral tissue-specific antigens (TSA) (101–103). mTEC express the transcription factors autoimmune regulator (AIRE) and forebrain expressed zinc finger 2 (Fezf2), which drive expression of a spectrum of TSA, including β cell-expressed proteins such as insulin (104–109). Thymic DC on the other hand acquire TSA and associated peptides through various mechanisms, including uptake of apoptotic mTEC, and trogocytosis of surface pMHC from mTEC (110–115). DC and B cells that traffick into the thymus also ferry TSA acquired from the periphery (116–120). SP expressing TCR with increased affinity/avidity for a given TSA-derived peptide are negatively selected and undergo apoptosis. A fraction of CD4^+^ SP expressing high affinity TCR for self-peptide, however, survive negative selection by differentiating into nFoxp3^+^Treg. SP that exhibit a low affinity for a TSA-derived peptide survive negative selection and egress from the thymus into the periphery (121). Noteworthy is that the efficiency of negative selection is limited early in ontogeny, thereby providing a discrete window of increased development of autoreactive T cells with enhanced affinity/avidity (122, 123). For instance, β cell autoimmunity develops in immunodeficient NOD.*scid* recipients reconstituted with T cells derived from transplanted thymic lobes of newborn but not 10 day or older NOD mice (124). Interestingly, autoimmunity has been reported in immunodeficient children with congenital athymia that receive an infant thymus transplant (125). The lack of structural organization of the medulla coupled with a largely immature mTEC pool expressing reduced TSA are thought to contribute to the limited efficacy of negative selection early in life.

### Factors That Impact the Specificity of the Anti-Self T Cell Receptor Repertoire Pool

The breadth and diversity of the TCR repertoire established during positive and negative selection reflects the TSA expressed and self-peptides processed and presented by cTEC and mTEC (98–100). Effective deletion of self-reactive SP for instance, is tightly linked to thymic TSA expression. In mice deficient of AIRE and Fezf2, mTEC fail to present a sufficiently broad constellation of TSA. Consequently, SP expressing anti-self TCR with increased affinity escape to the periphery and drive systemic autoimmunity (126). AIRE-deficiency also results in the failure of SP expressing TSA-specific TCR to differentiate into nFoxp3^+^Treg (127, 128). Humans with AIRE mutations develop Autoimmune Polyglandular Syndrome type-1 (APS-1) marked by multi-organ autoimmunity that includes T1D, Addison’s disease, and hypoparathyroidism (129, 130). Notably, the level of mTEC expression of insulin, a critical β cell autoantigen, can have a significant impact on the progression of T1D. Non-autoimmune prone C57BL/6 (B6) mice develop diabetes when mTEC lack insulin expression (131). Similarly, thymic insulin gene expression in humans is strongly associated with T1D susceptibility. Humans that have decreased variable number of tandem repeats (VNTRs) upstream of the insulin gene exhibit reduced thymic insulin expression, and increased T1D susceptibility (132, 133).

The efficiency of processing of self-antigens by cTEC and mTEC is also thought to play a key role in establishing the anti-self TCR repertoire (98, 100). Diabetes is prevented in NOD mice lacking thymus serine specific protease (TSSP) and Cathespin L (CatL), which are expressed by cTEC to generate MHC class II peptides (134–137). Lack of diabetes in TSSP- and CatL-deficient NOD mice correlates with an altered TCR repertoire expressed by CD4^+^ T cells, as well as increased thymic nFoxp3^+^Treg development. A modest decrease in diabetes is also seen in NOD mice lacking Cathespin S, which is involved in mTEC processing of MHC class II peptides (137). It is notable that TCR diversity is limited in CD4^+^CD25^-^ and CD4^+^CD25^+^ thymocytes from NOD (H2^g7^) versus B6 mice congenic for the H2^g7^ haplotype (138). Altered thymic antigen processing contributing to human T1D and myasthenia gravis is suggested by risk variants of TSSP and the CatL homolog *CTSL2* gene encoding CatV (139–141).

Finally, intrinsic properties of alleles of MHC molecules affect the repertoire of self-peptides presented in the thymus. As noted above the strongest genetic association with human T1D susceptibility is linked to specific alleles of the DQB1 gene; namely HLA-DQ8 (DQB1*03:02) and HLA-DQ2 (DQB1*02:01) (25–27). In NOD mice expression of the unique IA^g7^ class II molecule is needed for the development of β cell autoimmunity (142, 143). Notably, the DQ2/8 and IA^g7^ molecules share a common polymorphism at position 57 on the β chain which encodes a non-aspartic acid residue (144). This polymorphism favors the binding of peptides with a hydrophobic P9 anchor residue. In addition, IA^g7^-peptide complexes exhibit reduced surface half-life relative to other IA alleles (145). Therefore, T1D-associated MHC alleles are expected to promote sub-optimal presentation of self-peptides which enhances escape of β cell-specific SP with increased TCR affinity, while limiting the TCR repertoire of thymic Foxp3^+^Treg.

**Figure 1:**
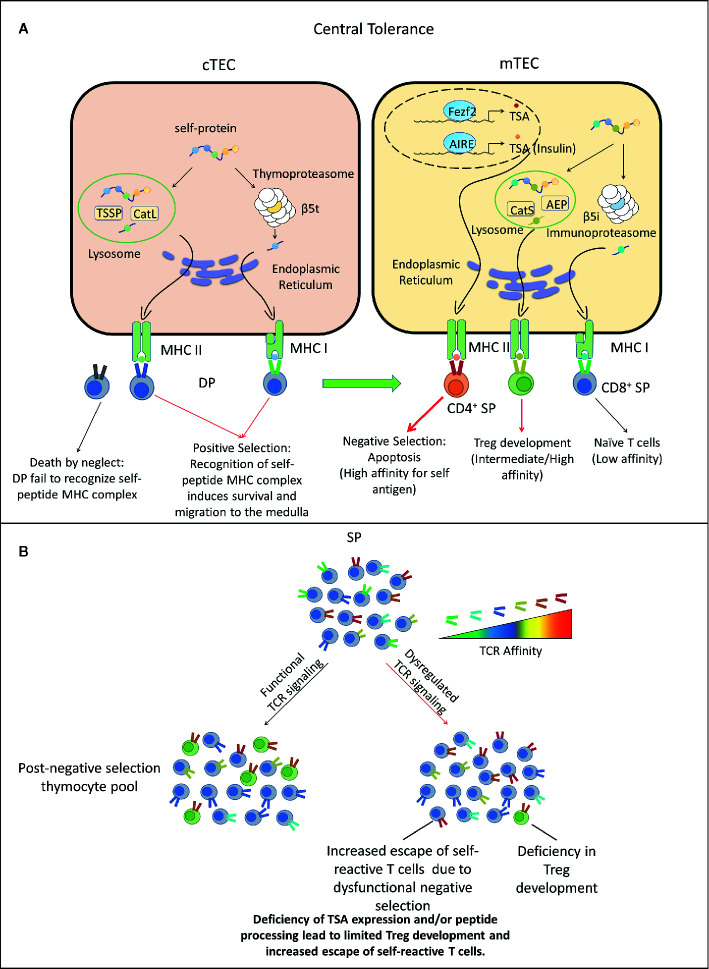
Dysregulated central tolerance impacts T cell receptor (TCR) signaling and establishes a T cell repertoire with increased type 1 diabetes (T1D) susceptibility. **(A)** In the thymus cortex, double positive thymocytes (DP) engage cortical thymic epithelial cell (cTEC) through TCR/peptide-MHC complexes (pMHC) interactions. Self-peptides are processed *via* the thymoproteasome and lysosomal proteases and displayed by MHC I and II molecules expressed by cTEC. DP recognizing these self-peptides receive survival signals, differentiate into CD4^+^ or CD8^+^ single positive thymocytes (SP) and migrate to the medulla. Alternatively, DP thymocytes incapable of transmitting TCR mediated survival signals die due to neglect. In the medulla, SP interact with mTEC or DC. Medullary TEC (mTEC) express a variety of tissue-specific antigens (TSA) such as insulin under the control of transcription factors autoimmune regulator (AIRE) and forebrain expressed zinc finger 2 (Fezf2). TSA are processed and presented on the MHC of mTEC, which can be acquired by DC by various mechanisms. SP with high TCR affinity for self-antigens are deleted through apoptosis, whereas SP exhibiting low TCR self-reactivity become naïve T cells. A fraction of CD4^+^ SP thymocytes with intermediate to high affinity differentiate into nFoxp3^+^Treg. Surviving SP exit the thymus and comprise the peripheral TCR repertoire. Red thick arrow: high TCR/pMHC affinity. Red thin arrow: intermediate TCR/pMHC affinity. Black arrow: low TCR/pMHC affinity. **(B)** Deficiency in self-peptide expression or processing by cTEC reduces TCR repertoire diversity and potentially increases susceptibility to β cell autoimmunity. Reduced expression and presentation of β cell-specific peptides by mTEC and other thymic APC limits: 1) negative selection leading to escape of an increased frequency of β cell-specific T cells with increased affinity/avidity, as well as impaired development of β cell-specific Foxp3^+^Treg. AEP, Asparaginyl endopeptidase.

In sum, thymic selection and central tolerance involve a complex set of events in which the expression and presentation of TSA-derived peptides impact: 1) the properties of the TCR repertoire expressed by T cells specific for self and foreign antigen, as well as 2) the development of protective nFoxp3^+^Treg. Dysregulation of these events can lead to an elevated peripheral frequency of autoreactive T cells expressing TCR with increased affinity, thereby enhancing susceptibility for pathogenic autoimmunity (**Figure 1**). The latter is further enhanced *via* peripheral processing and post-translational modification of islet antigens. *De novo* islet antigens such as hybrid insulin peptides (HIP) not presented in the thymus, stimulate the expansion of diabetogenic Teff in NOD mice (146–148). Interestingly, some HIP induce stronger TCR signaling than native proinsulin, suggesting low affinity/avidity autoreactive T cells can be readily activated by novel islet antigens (146, 147).

## Factors That Influence T Cell Receptor Signaling Strength During Type 1 Diabetes

### Efficient T Cell Activation Is Dependent on Signals 1, 2, and 3

TCR signaling involves a series of phosphorylation events initiated by the tyrosine kinase LCK-ZAP70 axis. LCK and ZAP70 activity is then propagated by secondary messengers including the PLC*γ*1, PI3K, MAPK, and Ca^++^ flux pathways (149–152). Over time, these signaling events culminate in epigenetic reorganization and gene transcription (93, 149, 153). Intrinsic and extrinsic factors regulate the nature, strength and functional outcome of TCR signaling in peripheral T cells. Engagement of the TCR with the peptide-MHC complexes (pMHC) forms an immune synapse (IS) between T cells and APC, which serves to regulate TCR signaling intensity and duration (154, 155). The IS facilitates interactions with the TCR and pMHC resulting in “signal 1” (154, 155). Optimal activation and differentiation of naïve T cells, as well as maintenance of Teff subsets, and differentiation of long-lived memory T cells (Tmem), however, requires additional signals (150). This includes “signal 2” delivered by T cell co-stimulatory molecules such as CD4, CD8, CD28, ICOS, and CD40L, and “signal 3” mediated by cytokines derived by T cells and APC (152).

### Anti-Self T Cell Receptor Signaling Is Required for T Cell Homeostasis

Preventing self-reactive TCR from exceeding the required signaling threshold between naïve T cell quiescence versus differentiation of pathogenic Teff after self-peptide recognition is a critical step to maintaining peripheral self-tolerance (151). This is particularly important since T cells continuously sample self-antigens *via* brief, low affinity TCR-pMHC interactions resulting in limited TCR-mediated phosphorylation events (156–158). This tonic TCR signaling is required for T cell survival and homeostasis. For instance, CD8^+^ and CD4^+^ T cells transferred into MHC class I and II deficient hosts, respectively, undergo accelerated apoptosis (159–162). Recent thymic emigrant (RTE) T cells also require tonic TCR signaling to fully develop into mature naïve T cells in the periphery. Therefore, the immune system exploits the intrinsic self-reactivity of TCR generated in the thymus to establish a balance between T cell homeostasis and unwanted autoimmunity through regulated T cell activation thresholds. This regulation is achieved partly through signaling events that are analog versus digital in nature (163). The proximal TCR-mediated phosphorylation of ZAP70 and upregulated gene expression of the transcription factor Nur77 are examples of analog signals that develop in a graded manner, directly reflecting the strength of TCR stimulus (163–165). In contrast, digital signaling involves events that are activated once a threshold is achieved with no intermediate state. Activation of NF-κB, a transcription factor driving T cell activation and function, is an example of TCR-mediated digital signaling (166). TCR analog events including Ca^++^-stimulated activation of IRF4 enable tonic signaling to occur below the threshold needed for efficient T cell activation (163, 167). In this way, despite low TCR reactivity to self, T cells sustain homeostatic gene expression without initiating an autoimmune response.

### Multiple Factors Influence T Cell Receptor Signaling Strength and Type 1 Diabetes Development

The strength of TCR signaling delivered upon recognition of a cognate, self-peptide-MHC complex is governed by a number of parameters including: TCR affinity, the frequency of pMHC, and the duration of the TCR-pMHC interaction (168). For any given antigen, the polyclonal TCR repertoire exhibits a spectrum of affinities (169). T cells expressing high affinity TCR can be activated at a relatively low pMHC frequency and *vice versa*. As alluded to above, the majority of autoreactive T cells are believed to express TCR with relatively low affinity that in turn permits escape of thymic negative selection. Accordingly, a high frequency of pMHC is required to activate this autoreactive T cell pool. On the other hand, inefficient thymic negative selection that is believed to occur in NOD mice, results in an autoreactive T cell pool expressing TCR with increased affinity (170–173). Therefore, a reduced frequency of pMHC is sufficient to stimulate and overcome the threshold for efficient activation of autoreactive T cells. The frequency of pMHC expressed by APC is dictated by a number of factors including the stability of the complex, which is in part influenced by peptide binding affinity and intrinsic properties of the MHC molecule. As noted above the IA^g7^ molecule has been reported to exhibit reduced stability which would be expected to limit T cell stimulation in the periphery (145). However, under proinflammatory conditions, APC upregulate antigen processing and presentation machinery as well as the expression level of co-stimulatory and MHC molecules that can compensate for the limited stability of IA^g7^ (174). One interesting scenario is that IA^g7^ instability reduces induction of T cell exhaustion, particularly during chronic antigen stimulation that occurs in the islets. Sustained TCR signaling promotes the formation of exhausted T cells (Tex), which serves to limit a given T cell response (175, 176). Levels of peptide-IA^g7^ complexes may be sufficient to stimulate Teff reactivity without driving exhaustion, thereby propagating chronic β cell autoimmunity.

Enhancing TCR signaling events in the periphery can break self-tolerance leading to autoimmunity. Polymorphisms in the protein tyrosine phosphatase, non-receptor type 22 (PTPN22) is associated with increased susceptibility to T1D and other autoimmune diseases (45, 46, 177–179). The role of PTPN22 in autoimmunity is complex, particularly since this tyrosine phosphatase is also expressed in B cells and DC, which contribute to the disease process (180, 181). In T cells, PTPN22 downregulates TCR signaling at multiple points including dephosphorylation and inactivation of the proximal kinases such as LCK and ZAP70 (179). PTPN22-deficient T cells exhibit increased LCK activation and Ca^++^ signaling (179). Furthermore, in NOD mice expressing the knock-in human PTPN22 risk variant, Teff and Tmem populations are increased, analogous to that seen in human subjects (182). This and other studies with a loss-in-function phenotype associated with the human PTPN22 risk variant consistently show increased T cell activation (179, 182). In this scenario, T cells expressing low affinity self-reactive TCR more readily overcome signaling thresholds needed for activation, expansion, and/or differentiation. Similarly, gene variants of PTPN2, another tyrosine phosphatase, have been associated with a T1D susceptibility gene (183). Human T1D risk variants have been identified that impact PTPN2 mRNA stability, protein structure, and expression levels (50, 184). β cell autoimmunity is exacerbated in NOD mice that selectively lack PTPN2 expression in T cells (185). Interestingly, enhanced disease progression is marked by an increased frequency of CD4^+^ Th1 and CD8^+^ Tc1 cells, as well as Tfh (185).

The strength of TCR signaling is also impacted by the CD28-CTLA-4 axis, which plays a role in T1D. A number of variants encoding CTLA-4 are associated with increased human T1D risk (47, 48, 186). Normally, CTLA-4 is upregulated shortly after T cell activation, competes with CD28 for binding to CD80 and CD86, and downregulates co-stimulatory “signal 2” (187, 188). The latter is achieved by recruitment of the phosphatases SHP-2 and PP2A to the cytoplasmic tail of CTLA-4. Activation of SHP-2 then inhibits LCK mediated phosphorylation of ZAP70 and activation of PI3K signaling pathways to block TCR signaling events. T1D risk variants are associated with reduced *CTLA4* mRNA levels and altered mRNA splicing resulting in a soluble isoform of CTLA-4 (47, 48, 186). Limited expression of CTLA-4 on the surface of conventional T cells (e.g. FoxP3^-^ T cells) is expected to readily promote expansion of Teff. Indeed, cancer immunotherapy trials have recently reported that CTLA-4 blockade induces T1D onset in some subjects (187). Furthermore, induced deletion of CTLA-4 in adult mice results in increased Teff numbers (189).

In addition to having a critical role in Teff subset differentiation, cytokine-mediated “signal 3” contributes to TCR signaling (152). IL-18 and IL-21 are particularly important in T1D. Deficiency in either IL-18 or IL-21 prevents the onset of diabetes in NOD mice (60, 190). IL-18 is known to stabilize Th1 responses, and in tandem with the TCR signaling related molecule Themis, enhances TCR signaling to self-antigens promoting T cell proliferation (190, 191). IL-21 is a key driver of B cell responses, but also enhances T cell proliferation of islet specific CD8^+^ T cells (63, 65, 192–196). Interestingly, IL-21 can mitigate development of Tex *via* STAT3 activation and the induction of the transcription factor BATF. TCR-induced IRF4 and IL-21 driven BATF cooperate to induce the expression of Blimp-1 that maintains Teff reactivity and blocks induction of exhaustion. IL-21 signaling has also been found to decrease expression of the Tex programmed cell death protein-1 (PD-1) (196). Therefore, IL-21 may help sustain β cell-specific T cell responses long-term through STAT3-regulated events, despite chronic TCR signaling. Interestingly, an intrinsic polymorphism increases STAT3 activation which is associated with severe multi-organ autoimmunity including T1D in humans (197). Approaches to attenuate pathogenic “signal 3” TCR events have been attempted in the clinic to alter T1D progression (198, 199). Specifically, an anti-IL-21 antibody therapy is currently being evaluated (NCT02443155) for efficacy in β cell preservation in newly diagnosed T1D patients.

## Impact of T Cell Receptor Signaling on the Molecular Landscape of T Cells

### T Cell Receptor Signaling and Regulation of Epigenetic Events

TCR signaling affects epigenetic and transcriptional regulation. A number of transcription factors are governed by TCR signaling and cytokine stimulation (**Table 1**), which shape the molecular landscape of naïve T cells transitioning into Teff, as well as the viability and expansion of Teff and differentiation of Tmem (200, 201). T cell activation induces changes in DNA methylation and acetylation, creating broad and lasting genetic modifications (200, 201). The most prominent markers denoting alterations in gene transcription are histone H3 lysine 27 trimethylation (H3K27Me3), and histone H3 lysine 27 acetylation (H3K27Ac). Methylation of histones such as H3K27Me3 have classically been associated with a closed chromatin state preventing gene transcription. On the other hand, acetylation markers such as H3K27Ac, correlate with an open chromatin state permissive for transcription. Accordingly, histone methyltransferases and deacetylases suppress gene expression, whereas histone acetyltransferases and demethylases promote gene expression. Enhancer of zeste homolog 2 (EZH2), the functional unit of the polycomb repressive complex 2 (PRC2), is a histone methylase that plays a key role in regulating various aspects of T cell immunobiology such a Foxp3^+^Treg stability (202, 203). Aberrant chromatin landscapes following T cell activation have been noted in numerous autoimmune diseases including rheumatoid arthritis, systemic lupus erythematosus, Grave’s disease, and T1D (204). Teff isolated from NOD mice display a unique chromatin structure conferring expression of T1D associated genetic loci (205).

**Table 1:** T cell receptor (TCR) signals influencing T cell subset differentiation and Teff function in type 1 diabetes (T1D).

T cell subset	TCR Signal Strength	Co-stimulatory Molecule	Cytokine Environment	Transcription Factor	Teff Cytokine	Teff Function
**Th1/Tc1**	Strong	CD28	IL-2, IL-12	T-bet	IFNγ	Involved in the defense against intracellular pathogens by lysing infected cells and inducing immune effector activation. Critical phenotype for the induction of T1D.
**Th17**	Moderate	–	IL-1β, IL-6, IL-21, IL-23, TGF-β	RORγt	IL-17A-E, IL-21, IL-22	Provide defense against extracellular pathogens by recruiting neutrophils and macrophages. Highly proinflammatory. Linked to T1D development.
**Tfh**	Strong	ICOS	IL-6, IL-21	Bcl-6	IL-4, IL-21	Support B cell activation and Ig affinity maturation in germinal centers. Tfh signatures are observed in both mouse and human T1D.
**Treg –Thymic/** **Adaptive**	Strong/ Weak	CD28	IL-2, TGF-β	Foxp3	IL-10, IL-35, TGF-β	Dampen T cell responses and prevent autoimmunity *via* contact dependent and independent mechanisms. Loss of function associated with T1D progression.
**Tr1**	Weak	Inhibitory Receptors	IL-27, TGF-β	Variable Foxp3 expression	IL-10	
**iTreg**	Weak	CD28	IL-2, TGF-β	Foxp3	IL-10, IL35, TGF-β	

The role of TCR signaling influencing epigenetic and transcriptional events is aptly seen with the transcription factor T-bet (206). T-bet expression is required for the development of β cell-specific pathogenic Teff in NOD mice (207). Expression of T-bet is regulated in an analog fashion directly reflecting the strength of TCR signaling (163, 208). High expression levels of T-bet drive the differentiation of short-lived type 1 Teff. T-bet has also been implicated in the differentiation of Tmem (209, 210). Intermediate TCR-induced expression of T-bet is needed for the generation of long-lived Tmem populations (209, 210). In addition to functioning as a transcription factor, T-bet remodels chromatin *via* recruiting histone demethylases such as JMJD3 (211). Here, T-bet induces transcriptionally permissive histone changes in promoters regulating expression of genes such as *CXCR3* and *IFNγ* (211). Thus, the magnitude of TCR signaling establishes expression levels of T-bet, which affects the phenotypic fate and migration of Teff. Similarly, reduced H3K27Me3 deposition is observed after TCR signaling (202). Here PI3K/AKT-mediated phosphorylation results in dissociation of EZH2 from promoter regions (202). This allows recently activated transcription factors to gain access to promoters exhibiting an euchromatin state primed for transcription. Notably the genetic changes established during activation of naïve T cells are preserved allowing for rapid recall responses of Teff and Tmem (212).

### Signals 2 and 3 Contribute to Shaping the T Cell Molecular Landscape

TCR signaling alone is typically insufficient to induce widespread chromatin changes and establish a given transcriptional profile. In addition to “signal 1”, co-stimulation of T cells *via* “signal 2” related molecules is needed to induce the full complement of gene expression during activation. For example, expression of IL-2 fails to occur when T cells are activated by TCR-alone (213). Co-stimulation by TCR and CD28 results in the acetylation and demethylation of the IL-2 promoter driving IL-2 expression (213). Interestingly, CD28 expression in NOD mice is critical to preserving self-tolerance during T1D (214). Here, reduced CD28 signaling is believed to contribute to poor IL-2 production compromising Foxp3^+^Treg function (214). Additionally, polymorphisms in NOD *Il2* can result in decreased IL-2 transcription also limiting the function and fitness of Foxp3^+^Treg (79, 215). Together, these findings suggest T cell activation signals work in concert to induce gene expression and maintain self-tolerance.

Cytokines produced by APC, as well as T cells in an autocrine or paracrine fashion, activate the STAT family of transcription factors that regulate Teff differentiation (**Table 1**) (209, 216). Specifically, cytokine mediated signaling and transcription through STATs can further upregulate expression of other T cell transcription factors, which both enhances and stabilizes the respective Teff phenotype (209, 216). Conversely the absence of essential cytokines can negatively impact the T cell response. In NOD mice, reduced T cell secretion of IL-2 generates a local *milieu* thought to promote chromatin remodeling and transcription that favors Teff by compromising the fitness and function of Foxp3^+^Treg (71, 85, 217–221). Similarly, in human T1D, polymorphisms in CD25 result in reduced FOXP3^+^ Treg sensitivity to local IL-2 (68). A diminished FOXP3^+^ Treg pool then further enhances the pathogenic β cell-specific Teff response.

## The Influence of T Cell Receptor Signal Strength on Expanded Effector T Cell Differentiation and Type 1 Diabetes Progression

### T Cell Receptor Signaling Strength and CD4^+^ Subset Differentiation

TCR signaling activates a common set of pathways, but the magnitude and quality of the events influence the outcome of Teff differentiation (93, 163, 222–224). Upon appropriate stimulation, naïve CD4^+^ T cells have the plasticity to differentiate into a number of distinct subsets based on pMHC frequency and/or cytokine environment (93, 222–224). Teff subsets have a signature transcription factor and secreted cytokine profile that establishes effector function (**Table 1**). Furthermore, specific Teff subsets require unique synergy of “signal 1, 2, and 3” for optimal differentiation and maintenance of Teff subsets. Lineage commitment is a pivotal aspect of autoimmunity; in multiple sclerosis (MS) for instance, increased disease severity is associated with Th17 reactivity (225–227). As previously mentioned, Teff in T1D largely exhibit Th1 and Tc1 phenotypes, although a role for Th17 and Tfh cells have been observed (51, 54, 64, 228, 229).

As noted above the strength of signal 1 can influence the differentiation of CD4^+^ T cells, dictating commitment toward Treg versus proinfammatory Teff (93, 222–224, 230). At low levels of TCR signaling, differentiation of various subsets of Treg, such as iFoxp3^+^Treg is favored (231, 232). In the case of iFoxp3^+^Treg for example, reduced signal 1 facilitates activity of the PTEN phosphatase, which blocks PI3K/AKT-mediated phosphorylation events, and in turn enables FoxP3 transcription driven *via* NF-κB, NFAT, and Foxo1 (233–236). Strong TCR signaling, however, antagonizes PTEN expression and activity, permitting PI3K/AKT signaling that inhibits FoxP3 transcription.

On the other hand, heightened signal 1 favors the differentiation of proinflammatory Teff such as Th1 cells. At relatively high TCR signaling threshold, T-bet expression is increased, which regulates expression of IFNγ and IL-12rβ chain (206, 237, 238). In an autocrine fashion IFNγ induces activation of STAT1, which then serves as a feed-forward loop promoting further T-bet transcription (224). IFNγ also acts on local APC to promote secretion of IL-12, which further promotes Th1 differentiation *via* STAT4 and increased T-bet and IFNγ expression (206, 237). T-bet and STAT4 also induce expression of the transcription factor Runx3, which enhances IFN*γ* expression and stabilizes the Th1 phenotype (206, 237, 239). Interestingly, islet-specific T cells from T1D patients have been found *in vitro* to have irregular T cell-APC immune synapse formation which favors low affinity TCR signaling. However, the increased antigenic density of the islets may promote robust TCR signaling required for progression of autoimmunity (240).

Strength of TCR signaling has also been reported to play a role in the differentiation of Th1 versus Tfh cells (93, 241, 242). Increased and chronic TCR signaling favors Tfh over Th1 differentiation (93). Elevated constitutive TCR signaling, along with T-bet, induces expression of Bcl-6 and the IL-2 receptor. At relatively reduced levels of TCR stimulation, IL-2 receptor activation of STAT5 induces IL-12 receptor and Blimp1 (241, 243, 244). These events lead to the suppression of Bcl-6, thereby permitting Th1 cell differentiation. However, increased TCR signaling uncouples the IL-2 receptor from the STAT5 signaling pathway, so that Bcl-6 is not suppressed, and Tfh cell differentiation progresses. Furthermore, signal 2 in Tfh cells through CD28 and ICOS play fundamental roles in preserving the Tfh phenotype during chronic immune responses such as murine and human T1D (241). One such mechanism is the chronic activation of ICOS on Tfh cells induces PI3K/AKT signaling that serves to repress the transcription factor Foxo1 which is a repressor of Bcl-6 expression and the Tfh lineage.

Co-stimulatory signals also can negatively regulate maintenance of the Th17 phenotype (245, 246). Previous studies have demonstrated that Th17 subset differentiation is impaired in the presence of combined TCR and CD28 signaling, resulting in: 1) activation of multiple negative regulators such as Bcl-6 and SOCS3, and 2) reduced sensitivity to the lineage skewing cytokines IL-1β and IL-2 (245, 246). In the absence of CD28 signaling, Th17 production of IL-17 is maximal whereas in the presence of CD28 signals T cells are diverted to a “Th1-like” IFNγ high producing population (246). These results suggest strong TCR signaling events skew toward Th1 lineage fates, and weaker TCR signals accompanied by the cytokine *milieu* result in Th17 commitment. Importantly, a role for Th17 cells has been associated with T1D (51, 66, 228). Th17 cells have been detected in the NOD mouse pancreas, and administration of blocking IL-17 antibodies reduced T1D onset in NOD mice (66). Overall, Th subset require distinct signaling conditions for specific lineage commitment.

### T Cell Receptor Signaling Strength and CD8^+^ Effector T Cell Differentiation

Analogous to CD4^+^ T cells, strength of signal 1 impacts the quality and magnitude of the CD8^+^ Teff response (163). Strong TCR signaling events in human and murine CD8^+^ T cells promote activation of inducible T cell kinase (ITK) that regulates the CD8^+^ T cell response by influencing the magnitude of Ca^++^ signaling. ITK activation controls the expression of transcription factors such as Blimp-1, T-bet and IRF4 that are needed to generate terminally differentiated CD8^+^ Teff capable of cytotoxic function (163, 247). Lack of Blimp-1, T-bet, or IRF4 upregulation impairs the function and expansion of short-lived Teff (163, 167, 248–255). Other properties which influence the pathogenicity of CD8^+^ Teff in the context of autoimmunity are also impacted by the strength of signal 1. Ovalbumin (OVA)-specific OT-1 CD8^+^ T cells primed with high but not low affinity altered peptide ligands (APLs) induce diabetes upon transfer into transgenic mice expressing OVA by β cells. The pathogenicity of these CD8^+^ Teff in part correlates with upregulation of the integrin very late antigen-4 (VLA-4), which facilitates islet infiltration (256).

## The Role of T Cell Receptor Signaling in the Maintenance of Effector T Cell Function and Type 1 Diabetes Progression

### T Cell Receptor Signaling in Effector T Cell Function and Memory T Cell Development

TCR signaling is critical for the maintenance of T cell viability, effector functions, and tissue residency (163). Inhibition of TCR signaling in NOD mice by FK506 treatment or blockade of the CD4 and CD8 co-receptors by monoclonal antibodies both prevent the progression and induce remission of T1D (257–259). Here, the diminution of TCR signaling in islet resident T cells promotes islet egress and trafficking back into circulation (257–259). This results from reduced IFNγ production by islet Teff, which in turn reduces the production of CXCL9/10 causing a failure in islet T cell retention (257–259). These findings highlight how TCR signaling directly affects Teff cytokine production and tissue residency critical to the progression of T1D.

The long-term survival and function of T cells is also a requirement for the development of T1D (260, 261). Typically, long-lived T cell responses are carried out by Tmem. An increased precursor frequency, rapid cytokine production, and the expression of unique trafficking molecules characterize Tmem responses (209, 216). The precise role of Tmem in autoimmunity is difficult to define since anti-self-reactivity is chronic due to the constitutive presentation of autoantigens. However, it is likely that a Tmem-like phenotype is necessary to sustain persistent autoreactivity. Indeed, GAD65-specific CD4^+^ T cells marked by IFNγ secretion and a capacity to infiltrate and destroy human islets transplanted in mice are detected over numerous years in human T1D patients (262–264).

TCR signaling plays a fundamental role in the development of Tmem cells (150). Both strong and weak TCR signals induce Tmem (208). Studies suggest that the quality and duration of TCR signaling coupled with the activation of specific transcription factors such as IRF4 influence Tmem formation (150, 209, 216). Increasing TCR signaling strength directly induces IRF4 expression with high IRF4 levels resulting in Teff and intermediate IRF4 expression establishing Tmem (167, 247, 255). Mice deficient in IRF4 expression fail to develop fully functional Tmem, and lack of IRF4 expression results in Tex (265, 266). Strikingly, NOD mice deficient in IRF4 fail to develop T1D and show dysfunctional T cell responses (267). Maintenance of long-lived Tmem responses has been tied to the pro-memory transcription factor TCF1 (268–270). High expression of IRF4 inhibits TCF1 expression showing that TCR signaling controls the maintenance of Teff responses (265). Recent work has suggested the long-lived β cell-specific responses are maintained *via* TCF1 which sustains the autoimmune response in human T1D (260). Furthermore, transplantation of pancreatic islets into diabetic mice leads to recurrent T1D and transplanted islet destruction (271). These results indicate a long-lasting autoreactive T cell population exists after self-antigen clearance.

### T Cell Receptor Signaling and the Formation of Exhausted T Cell and Type 1 Diabetes

The clearance of antigen also appears to be an important step in the development of Tmem. Failure to clear antigen in a timely fashion leads to a state of chronic inflammation which negatively impacts the formation of Tmem in studies comparing acute versus chronic lymphocytic choriomeningitis virus (LCMV) infection (272). Sustained TCR signaling initially favors Teff development, but then leads to the formation of Tex (175, 176, 188). This continued signaling and proinflammatory cytokine production can suppress the expression of pro-memory genes such as Foxo1 (273). Tex have been reported in several immune models of infection, cancer, and autoimmunity with both CD4^+^ and CD8^+^ T cells susceptible to developing an exhausted phenotype as chronic TCR signaling is a hallmark of these conditions (175, 176, 188).

A key exhaustion marker that has been associated with a Tex phenotype in multiple diseases is PD-1 (175, 176, 188, 272). Interestingly, functional inhibition of this molecule has significant consequences on autoimmunity (175, 176, 272, 274). Phenotypically, Tex exhibit a reduced Teff capacity due to the upregulation of molecules associated with dampening TCR signaling events (275). PD-1 is upregulated rapidly in an analog fashion following TCR stimulation. Chronic TCR signaling, however, results in permanent upregulation of PD-1 expression, and T cell dysfunction (175, 176, 272, 275). Inhibition of TCR signaling occurs upon PD-1 binding to PDL-1 or PDL-2 expressed by APC or the targeted tissues (275). Ligand binding results in phosphorylation of PD-1 by LCK, and recruitment of the tyrosine phosphatase SHP-2, which in turn negatively regulates phosphorylation of CD28 (275, 276). The latter limits Zap70 and PI3K signaling to inhibit TCR-dependent Teff functions (275, 276). This continued expression of PD-1 then drives T cells into a terminally exhausted state (175, 176, 272, 275).

The PD-1-PDL-1/2 axis is a prominent regulatory pathway in autoimmune diseases such as T1D (274, 277–281). PD-1 deficient NOD mice develop aggressive T1D, as do NOD mice treated with an anti-PD-1 antibody (277, 278). A subset of human cancer patients receiving check-point blockade including anti-PD1 antibody have also been reported to develop autoimmune diseases including T1D (187). In addition to PD-1, there are a number of other factors associated with the Tex phenotype including cell surface proteins such as CTLA-4, LAG3, and TIM-3 which further dampen TCR signaling and cellular metabolism suppressing Teff function (175, 176, 188, 272). NOD mice deficient in or receiving blocking antibodies to these molecules results in acceleration of T1D similar to the disruption of PD-1 function (282–284). A diminished frequency of Tex correlates with the progression of human β cell autoimmunity (279, 285). The latter is consistent with T1D subjects exhibiting an increase in Tfh, which produce IL-21. Notably, IL-21 blocks the induction of T cell exhaustion (196). Interestingly, anti-CD3 therapy has been found to induce a Tex cell phenotype in both NOD mice and human T1D patients (280, 281, 286). These results indicate that inducing and maintaining Tex are a key component of T1D progression. How TCR signaling and the cytokine *milieu* during T1D limits the transcriptional development of terminally exhausted autoreactive T cells continues to be an area of study.

## Conclusion

**Figure 2:**
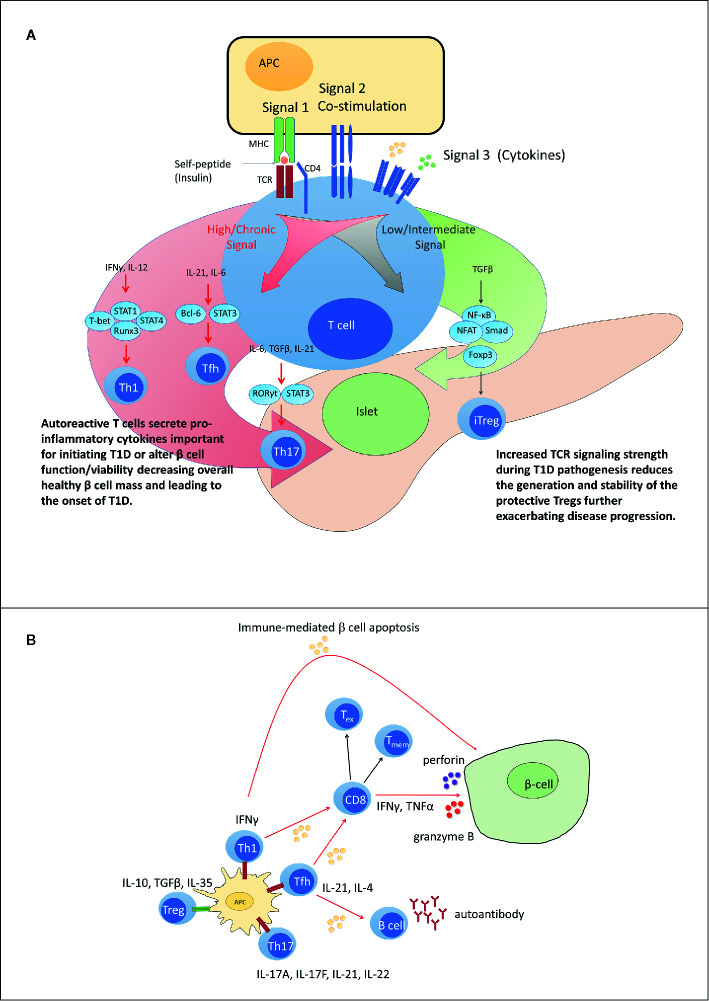
Dysregulated peripheral tolerance and the role of T cell receptor (TCR) signaling in type 1 diabetes (T1D) pathogenesis. **(A)** Upon interacting with antigen presenting cell (APC), peripheral T cells differentiate into distinct subsets through activation of specific sets of transcription factors based on TCR signal strength (signal 1), co-stimulatory molecule engagement (signal 2) and the cytokine environment (signal 3). Low TCR signaling favors differentiation of adaptive regulatory T cell subsets such as iFoxp3^+^Treg. Strong and/or persistent TCR signaling favors differentiation of Th1, Tfh, and Th17 subsets. Increased TCR signaling in T1D aids in disrupting the balance between pro inflammatory and regulatory immune responses. **(B)** In T1D, increased affinity for and/or prolonged interaction with β cell peptide-MHC complexes results in elevated TCR signaling. Elevated TCR signaling coupled with signals derived *via* co-stimulation and proinflammatory cytokines, promotes differentiation of pathogenic Teff such as IFNγ-producing Th1 cells and cytolytic Tc1 cells, that migrate into the islets and mediate β cell destruction. Chronic TCR signaling normally leads to T cell exhaustion and dampened Teff function. However, elevated levels of IL-21 rescue Teff from terminal exhaustion to maintain β cell destruction. Tmem contribute to maintenance of β cell autoimmunity by providing a source of chronic proinflammatory cytokines upon antigen stimulation. Red TCR (high TCR signal). Green TCR (low TCR signal).

The disease process of T1D is complex and heterogenous, influenced by genetic and ill-defined environmental factors. Multiple immune defects contribute to a failure of β cell-specific self-tolerance which impacts the function of various immune effector cells, including T cells. Genetic variants associated with increased T1D susceptibility have been linked to the generation of self-reactive TCR and dysregulated TCR signaling events. Evidence indicates that alterations in signals 1, 2, and 3 lead to impaired central and peripheral tolerance inducing mechanisms (**Figure 2B**). The latter culminates in β cell autoantigen-induced T cell activation, expansion, and subset differentiation driven by epigenetic and transcriptional outcomes that promote a proinflammatory response (**Figure 2A**). We propose that signal 1 is the dominant TCR signaling event driving disease progression, consistent with the fact that the strongest genetic association exists between specific HLA alleles and T1D susceptibility. The greatest impact of signal 1 is expected to be in the thymus, which establishes the repertoire specificity, affinity, and frequency of the anti-self TCR pool, as well as development of nFoxp3^+^Treg. On the other hand, dysregulation of signals 2 and/or 3 serve to modify signaling outcomes in peripheral β cell-specific T cells that favor differentiation and maintenance of pathogenic Teff and Tmem, while limiting terminal Tex cell development and a protective FOXP3^+^Treg response. The degree of dysregulation within the respective TCR signaling pathways may influence the heterogeneity observed in the pace of disease progression, the frequency of T cell infiltration of the islets, and the age of T1D onset in humans.

Although TCR signaling defects are associated with mediating β cell autoimmunity, a better understanding of those alterations may also provide clues on how to design effective individualized treatments. The targeting of one or multiple TCR signaling events offers one strategy to modulate the differentiation and activity of autoreactive Teff for the purpose of T1D prevention and treatment. Indeed, varying degrees of efficacy have been seen in preclinical and clinical studies targeting TCR signaling pathways *via* pharmacological inhibitors, β cell-derived peptides and APLs, recombinant cytokines, monoclonal antibodies and fusion molecules (287–289). Future work is needed to focus on the targeted delivery of TCR modulating therapeutic agents directly to autoreactive T cells, thereby selectively dampening the autoimmune responses and preserving protective acquired immunity.

## Author Contributions

All authors contributed to the article and approved the submitted version.

## Funding

This work was supported by the National Institutes of Health grants R01DK100256, R01AI139475, R01AI141631, R21AI115752 (RT), and T32AI007273 (MC), and the American Diabetes Association 1-18-PDF-108 (MC).

## Conflict of Interest

The authors declare that the research was conducted in the absence of any commercial or financial relationships that could be construed as a potential conflict of interest.
